# Modern Sociology

**Published:** 1903-02-07

**Authors:** 


### MODERN SOCIOLOGY.

### INSURANCE AND CRIME.

No man can honestly make money out of an insurance policy. This is the thesis with which Mr. Alexander Colin Campbell starts his volume on Insurance and Crime (Putnam's). A man who insures his house or furniture or other possessions against loss by fire for only as much as they are worth, gets, if the thing insured is destroyed only, at most, the sum insured for minus the sum paid in premiums. He makes no profit out of the transaction. But if he insure his property for more than it is worth, he stands to gain, if it is destroyed, a profit to the extent, minus premiums, of the over-insurance. Herein lies the temptation to fraud. A shopkeeper on the verge of bankruptcy insures his stock for far more than it is worth, and then a convenient fire occurs, and in place of a moribund business and unsaleable stock, he receives money in hand which will clear his liabilities and perhaps start him afresh. A shipowner has an old vessel, unseaworthy and worth but little for breaking up. But if he can insure her for a good sum, he does not mind if she goes down on her voyage. In fact her wreck may pay him better than her safe return. It is true that lives may be lost by the fire and sailors drowned when the coffin ship goes down, but it is not doing human nature too much wrong to say that here and there are persons to whom the possible risks to others will not outweigh the certain gain to themselves. That is stating the case as mildly as possible, and those who read Mr. Campbell's book will find ample proof for the contention.

Of course the insurance companies who have to pay the damages when loss occurs should take good care that these over-insurances are not permitted. It is surely to their interest to see that an insurance risk is not to all intents and purposes a certainty. They should, but they don't. In the first place they, so to speak, pool their risks, and as there are always a majority of people who don't want to have their property destroyed, and whose earnest prayer is that they may not need to ask for the insurance-money, the profit from these covers the loss on the others.

Life insurance stands upon a different footing from the insurance of ships against loss or buildings against fire. In these the companies are taking chances which may never occur, and may reasonably be more venturesome than when they are actually promising a sum to be paid in the certain event of the insurer's death. They are bound sooner or later to be obliged to pay the money to the insurer's representatives, and their profit can come only from those lives which last long enough to pay more than the sum insured for. Therefore the companies demand not only that the life should be sound, but that the person who insures should have "insurable interest" in the life insured. A man can easily insure his own life in favour of his wife and children, but he would have some difficulty in insuring the life of the wife. The one insurance is a reasonable provision for those dependent on him, but why should he expect to gain by the death of his wife? It will not affect his earning capacity. And if such an insurance was effected, and the wife died soon after, there would be every chance of the company refusing to pay the sum insured for, and demanding an inquiry into the cause of death. The limits within which life insurance is reasonable and permissible are fairly clear, and yet the history of this branch of the subject can show a long and terrible record of crime.

For much of this the insurance companies have had themselves to blame, though being bit frequently has made them careful in making sure of the legitimate insurable interest of the insurer in the life insured. It is hardly likely that such a man as Thomas Griffiths Wainewright would now be able to carry on his career of murder as long as he did, apparently with but little trouble, in the twenties of last century. Wainewright, a clever though superficial writer, a friend of Macready, of Sir David Wilkie, and of Charles Lamb, lived regularly beyond his income. He began his criminal career by forgery, in order to obtain possession of the principal of a small fortune, the income of which he enjoyed. When the money thus obtained was spent, he and his wife went to live with a wealthy uncle, whose heir he was. Within a year the uncle died suddenly. Wainewright then induced his wife's mother and half-sisters to share his home. The mother also died suddenly. Then Wainewright insured one of the sisters-in-law, "a buxom, handsome girl of 20," for the enormous sum of £18,000, and this being done he poisoned her. The insurance companies refused to pay on the ground that Wainewright had no insurable interest in the girl's life. Which was true enough, only the companies ought to have attended to the point before they granted the policies of insurance. Had they done so the poor girl's life might have been spared. Occasionally a man or woman is murdered for the sake of the insurance on their life even when the insurable interest is a reasonable one, but when a man insures the life of a young and healthy sister-in-law in his own favour there is ground for suspecting at least the possibility of foul play.

Besides actual murders there have been endless frauds connected with life insurance. A needy man may sell his insurance policy. As the purchaser has to continue paying the premiums during the life of the insured, he does not, cannot, desire that life to be a long one. If he does not murder the original owner of the policy, he may palm off some dying man as being he, and when the latter dies have him buried in the name he desires, and claim the insurance money. Such arrangements can occasionally be made at the cost of only a little money given to the moribund personator or his heirs. Long ago in one of the magazines appeared a story in which two adventurers tried a trick like this with an insurance policy which they had bought cheaply, and engaged a young man, apparently in the last stages of consumption, to personate the man who had taken out the policy, promising him that he should have every comfort during the remainder of his life. But, like Charles II., the consumptive was "an unconscionable time in dying," and at last it comes out that he is the "Living Skeleton" of a travelling show, who had thus managed to obtain a luxurious holiday, but finally, when he had had enough of rest, threw up his job, and left the speculators in insurance lamenting.

The law has intervened to put an end to the horrors of child insurance, a thing which began in the simple and natural desire of people whose incomes would stand no sudden extra strain, to avoid the disgrace of a "parish burial" if any of their children should die. In the end it developed into a scheme by which heartless parents or guardians made money by dooming helpless children to death by starvation and neglect.

A fruitful source of all fraudulent insurance is the custom of the less scrupulous sort of companies of paying their agents by commission on the business done. The more lives the agent insures, the more commission he receives, and, therefore, he is not too inquisitive about the lives offered to him, nor about the reasons given for taking out a policy. That is none of his business. But it is the business of the company which will finally have to pay the insurance money, and which loses on every early death. And it is the business of the honest insurer who has to pay a higher rate of premium because poor lives are accepted and the acceptance of an interest in the life insured which is not legitimate may lead to sudden and mysterious deaths.

